# Outcomes of H3K27M-altered diffuse midline glioma adult Chinese patients and real-world experience with German-sourced ONC201 targeted therapy: A multi-center study in Hong Kong

**DOI:** 10.1093/noajnl/vdag074

**Published:** 2026-03-21

**Authors:** Peter Y M Woo, Clara K W Lee, Lai-Fung Li, Victor K H Hui, Danny T M Chan, Michael W Y Lee, Tony K T Chan, Desiree K K Wong, Joyce S W Chow, Jason M K Ho, Jason K H Chow, Teresa P K Tse, Natalie M W Ko, Ka-Man Cheung, Angus K C Leung, Kenneth C W Wong, Herbert H F Loong, Wai-Sang Poon, Aya El-Helali

**Affiliations:** Department of Neurosurgery, Prince of Wales Hospital, Hong Kong; Otto Wong Brain Tumour Centre, The Chinese University of Hong Kong, Hong Kong; Department of Neurosurgery, Princess Margaret Hospital, Hong Kong; Department of Neurosurgery, Queen Mary Hospital, Hong Kong; Department of Neurosurgery, Prince of Wales Hospital, Hong Kong; Otto Wong Brain Tumour Centre, The Chinese University of Hong Kong, Hong Kong; Department of Neurosurgery, Prince of Wales Hospital, Hong Kong; Otto Wong Brain Tumour Centre, The Chinese University of Hong Kong, Hong Kong; Department of Neurosurgery, Princess Margaret Hospital, Hong Kong; Department of Neurosurgery, Queen Elizabeth Hospital, Hong Kong; Department of Neurosurgery, Queen Elizabeth Hospital, Hong Kong; Department of Neurosurgery, Kwong Wah Hospital, Hong Kong; AMO Oncology Centre, Hong Kong; Department of Clinical Oncology, Prince of Wales Hospital, Hong Kong; Department of Clinical Oncology, The Chinese University of Hong Kong, Hong Kong; Department of Neurosurgery, Prince of Wales Hospital, Hong Kong; Otto Wong Brain Tumour Centre, The Chinese University of Hong Kong, Hong Kong; Department of Clinical Oncology, The University of Hong Kong, Hong Kong

**Keywords:** dopamine receptor antagonist, dordaviprone, H3K27M-mutant/altered diffuse midline glioma, imipridone, ONC201, overall survival

## Abstract

**Introduction:**

A novel dopamine-receptor small molecule inhibitor, ONC201, was observed to elicit a treatment response in patients with H3K27M-altered diffuse midline gliomas (DMG). Given restricted access to this therapy, an alternative formulation from Germany (GsONC201) was made available by compassionate means. We describe the treatment outcomes of these patients and our experience with GsONC201.

**Methods:**

This was a multicenter retrospective study of adult Chinese patients with histologically confirmed H3K727M-altered DMG. The primary endpoint was overall survival (OS). Secondary endpoints were progression-free survival, the observed response rate (ORR) at 3 months after radiotherapy and GsONC201-associated adverse effects.

**Results:**

Twenty-seven patients, median age of 40 years (range: 31-52), were identified. Fifty-two percent (14/27) of tumors arose from the thalamus followed by the pons (22%, 6). Eighty-five percent (23/27) of patients received standard-of-care (SOC) fractionated radiotherapy. Thirty-seven percent (10/27) of patients received GsONC201 of which 80% (8/10) started it as first-line monotherapy after SOC. The mOS of the entire cohort was 17.4 months (IQR: 12.1-30.0). GsONC201 + SOC patients (8) had a mOS of 18.9 months (IQR: 11.3-54.4) compared to 16.0 months (IQR: 12.5-27.6) for SOC-alone patients (13, *P* value: .57). The ORR was 33% (7/21) and 63% of GsONC201 + SOC patients had a treatment response compared to 15% of SOC-alone patients (*P* value: .01). No GsONC201-associated adverse effects were observed.

**Conclusion:**

This is the first real-world study to review the outcomes of first-line imipridone-class agent therapy in adult Chinese DMG patients. GsONC201 was well-tolerated, but its effect on OS remains unknown.

Key Points

- H3K27M-altered DMG is a rare disease with a crude incidence 0.04 per 100 000 population.
- GsONC201 was a well-tolerated first-line oral targeted agent.
- A higher proportion of patients that had standard-of-care (SOC) therapy and first-line GsON201 experienced an MRI observable treatment response compared to those that received SOC therapy alone.
- Long-term complete disease remission was observed in certain patients that received first-line single-agent GsONC201 after radiotherapy.

Importance of the Study

There is no widely-accepted systemic oncological treatment for patients diagnosed with H3K27M-altered diffuse midline glioma (DMG). The only standard-of-care treatment modality is radiotherapy. A number of studies have described the positive overall survival (OS) outcomes of patients treated with an investigational orally-active, first-in-class imipridone, a small molecule selective D2 dopamine receptor antagonist known as ONC201. An alternative formulation of this agent produced in Germany, with comparable pharmacokinetic and pharmacodynamic effects, German-sourced ONC201 (GsONC201), is being increasingly prescribed for patients without clinical trial access. This study reviewed the treatment outcomes of Chinese adult H3K27M-altered DMG patients and our experience with this novel targeted therapeutic agent. The observed response rate of patients prescribed first line GsONC201 after radiotherapy was significantly higher than those that received radiotherapy alone. No drug-associated adverse effects were documented. For certain patients, a sustained complete response to GsON201 according to Response Assessment in Neuro-oncology criteria was observed.

H3K27M-altered diffuse midline glioma (DMG) is a rare WHO grade 4 primary CNS tumor that was first recognized as a distinct diagnosis by the fourth WHO Classification in 2016.^1^ Its incidence is 0.06 per 100 000 population predominantly affecting pediatric and young adults at a median age of 15 years.^2^ The tumor is characterized by an epigenetic histone mutation resulting in the absence of histone 3 trimethylation and the substitution of lysine 27 (K27) by methionine (K27M), i.e. H3K27me3 loss.^3^^,^^4^  *H3K27M* mutations are observed in 15-60% of adults with DMG.^5^ Due to their deep-seated, frequently eloquent locations, these tumors are generally not amenable to surgical resection and standard-of-care (SOC) is limited to fractionated radiotherapy (RT) that confers a modest overall survival (OS) of only 9-11 months.^5^^,^^6^

There is a growing body of evidence supporting the clinical efficacy of ONC201 (dordavirpone, Jazz Pharmaceuticals plc, Dublin, Ireland), an orally active, first-in-class imipridone, small molecule selective D2 dopamine receptor (DRD2) antagonist for both newly diagnosed and recurrent H3K27M-altered DMG.^7–9^ The agent inactivates Akt/ERK signaling to induce tumor cell apoptosis through the TNF-related apoptosis-inducing ligand pathway.^10^^,^^11^ It also exerts its action as an agonist of caseinolytic protease P (ClpP) that drives mitochondrial respiratory chain enzymes to trigger an integrated tumor stress response.^12^ Several prospective clinical studies, including a multinational randomized, placebo-controlled trial (RCT), are currently underway (clinicaltrials.gov identifier: NCT05580562).^13^ An alternative formulation of ONC201, sourced from Germany (GsON201), with similar pharmacokinetic and pharmacodynamic characteristics, was found to have comparable biological on-target activity, with encouraging clinical outcomes.^14^^,^^15^ Hong Kong residents were able to individually source GsONC201 through a German compassionate prescription program. This study aimed to determine the incidence of H3K27M-altered DMG among adult Chinese patients and identify the patterns of care and their treatment outcomes especially with regard to GsONC201.

## Methods

This was a territory-wide, multicenter retrospective review of consecutive Chinese adult patients (≥18 years) that was approved by the Hospital Authority (HA) institutional review board (reference number: KC/KE-18-0262/ER-4). Hong Kong is a coastal city situated in South-East China that provides universal public healthcare to 7.5 million residents. The HA is responsible for managing > 90% of inpatient bed-days in the territory with a comparably high rate of follow-up. Patients were identified from the Hong Kong High-Grade Glioma Registry, a centralized clinically annotated database of consecutively documented histologically confirmed high-grade glioma adult patients managed by the city’s public healthcare system.^16^ Patients diagnosed with H3K27M-altered DMG according to the fourth WHO Classification of CNS Tumors from January 1, 2016 to October 30, 2024 were reviewed. All examined tumor tissue was formalin-fixed paraffin-embedded. H3K27M and H3K27me3 immunohistochemistry (IHC) or Sanger sequencing to detect K27M mutations of *H3F3A/B* were performed. First-line SOC was defined as fractionated RT of 54-60 Gy administered 5 days/week (1.8-2.0 Gy/fraction) for 30 fractions. Hypofractionated RT regimens of 40 Gy in 15 fractions or 34 Gy in 10 fractions were also considered SOC.^17^^,^^18^ For patients that received TMZ, it was concomitantly prescribed with RT (CRT) at a dose of 75 mg/m^2^ daily for six weeks and subsequently at 150-200 mg/m^2^ daily for five days every four weeks until tumor recurrence or if intolerable adverse effects occurred. None of the patients were enrolled in clinical trials.

For patients prescribed GsONC201, the agent was administered orally after RT, 625-680 mg, weekly, two hours before or after meals. Patients were advised to avoid consuming high-calcium foods or beverages for four hours before and after administration. They were assessed monthly for treatment-associated adverse effects, with peripheral blood sampling performed to evaluate complete blood counts, renal function, and liver function. Patients were regularly scanned with gadolinium contrast-enhanced MRI every three to six months. All scans were assessed according to the Radiological Assessment in Neuro-oncology 2.0 (RANO) and Response Evaluation Criteria in Solid Tumors (RECIST) 1.1 criteria.^19^

Data were broadly classified into patient-related, disease-related, and treatment-related. Patient-related data included functional performance upon presentation according to the Eastern Cooperative Oncology Group (ECOG). Disease-related data included location of the tumor, its molecular characteristics, and the presence of obstructive hydrocephalus. Treatment-related data included the use of RT, chemotherapy, targeted therapy agents, such as GsONC201, and the need for cerebrospinal fluid shunting. Extent of resection (EOR) was broadly classified into biopsy, subtotal resection (STR), and gross total resection according to the neurosurgeon’s intraoperative assessment. A review of second-line treatment was also performed and the duration of its initiation, defined as the date of MRI detection of tumor recurrence to the starting of salvage therapy, was recorded.

The primary study endpoint was overall survival (OS), defined as the time duration from the date of the operation that confirmed the histological diagnosis till the date of death. In particular, the median OS of patients prescribed first-line GsONC201 after RT (GsON201 + SOC) was compared to those that only received RT (SOC-alone). Secondary endpoints were progression-free survival (PFS), defined as the time interval from the date of the operation to the date of radiological tumor recurrence and the proportion of patients achieving RANO 2.0-defined categories at three months post-RT. At this timepoint, the observed response rates (ORR), defined by the RANO 2.0 and RECIST 1.1 criteria were recorded. For GsONC201 + SOC patients, the duration required to detect a response, i.e. from the date of starting the targeted therapy to the date of MRI-detected observed response, was also documented. GsONC201-related adverse effects were documented and graded according to the National Cancer Institute’s Division of Cancer Treatment and Diagnosis.^20^ Follow-up duration was defined as the time interval from the date of the operation to either the date of last clinic follow-up or the date of death. Patient data were censored by 20 January 2026.

### Statistical Analysis

Demographic cohort data were summarized using standard descriptive statistics. To test differences between groups, the Pearson’s chi-squared test (categorical variables), two-tailed Student’s *t*-test for independent groups (continuous variables) and one-way ANOVA was carried out for continuous variables with more than two groups. A *P* value < .05 was considered statistically significant. Survival probabilities for the entire cohort were represented by Kaplan-Meier plots and RECIST 1.1 tumor size changes from baseline were summarized in a waterfall plot. These analyses were performed utilizing the Statistical Package for the Social Sciences software version 21.0 (SPSS Inc.).

## Results

### Patient, Tumor, and Treatment Characteristics

Twenty-seven adult Chinese patients were diagnosed with H3K27M-altered DMG during this nine-year review period. The crude incidence in Hong Kong was 0.04 per 100 000 population. All tumor specimens were immunopositive for H3K27M. 47% (11/27) specimens had additional *H3F3A* Sanger sequencing and all detected a positive c.83A>T, p. Lys28Met heterozygous missense mutation. All tumors were isocitrate dehydrogenase-1 (*IDH-1*) wildtype and promoter methylguanine methyltransferase (*MGMT*) unmethylated. Nineteen (70%) patients had their tumors subject to ATRX IHC testing of which 21% (4/19) had loss of expression. Thirteen (48%) of tumors underwent IHC *TP53* mutation testing of which 69% (9/13) were immnunopositive.

The median age of diagnosis was 40 years (range: 31-52) and the female-to-male ratio was 1:1.5 (Table 1). The median duration of follow-up was 13.8 months (range: 1.1-72.9). Seven percent (2/27) of patients were lost to follow-up. Patients had a median preoperative ECOG functional performance of 2 (range: 0-3). Half of the tumors were located in the thalamus (52%, 14/27), followed by the pons (22%, 6) and the hypothalamus (11%, 3). Twelve patients (44%) required ventriculo-peritoneal shunting for hydrocephalus within one month of diagnosis, and ultimately, 15 patients (56%) required shunting during their entire disease course. Forty-eight percent (13/27) of patients underwent a tumor biopsy and the remaining had STR.

**Table 1.** Characteristics of H2K7M-mutant DMG patients: comparison of SOC-alone patients *versus* GsONC201 + SOC patients

Characteristics	Entire cohort	SOC-alone	GsONC201 + SOC*	*P* value
	*n* = 27 (%)	*n* = 13 (%)	*n* = 8 (%)	
Patient factors				
Age, years, median (IQR)	40 (31-52)	41 (32-54)	46 (31-52)	.55
Age < 60 years	24 (89)	11 (85)	8 (100)	.47
Male	11 (41)	8 (62)	2 (25)	.09
Preoperative ECOG				
median (IQR)	2 (1-2)	2 (1-2)	2 (1-2)	1.000
0 or 1	13 (48)	6 (46)	5 (63)	.08
Tumor factors				
Location				
Thalamus	14 (52)	8 (62)	4 (50)	.66
Brainstem	9 (33)	3 (23)	3 (38)	.57
Hypothalamus	3 (11)	2 (15)	1 (13)	.89
Hydrocephalus on presentation	12 (44)	7 (54)	3 (38)	.25
Treatment factors				
EOR				
Gross total resection	0	0	0	-
Subtotal resection	14 (52)	6 (46)	4 (50)	.88
Biopsy	13 (48)	7 (54)	4 (50)	.77
VP shunting	15 (56)	8 (62)	4 (50)	.25
RT	23 (85)	13 (100)	8 (100)	.10
TMZ CRT	17 (63)	10 (77)	7 (88)	.26
First-line GsONC201	8 (30)	-	8 (100)	-
≥3 months	8 (30)	-	7 (88)	-
≥6 months	5 (19)	-	4 (50)	-
Bevacizumab upon recurrence	7 (37)	4 (31)	3 (38)	.37

CR, complete remission; CRT, chemoradiotherapy; ECOG, Eastern Cooperative Oncology Group; EOR, extent of resection; GsONC201, German-sourced ONC201; OS, overall survival; PD, progressive disease; PFS, progression-free survival; PR, partial remission; RANO; radiological assessment in neuro-oncology; RT; radiotherapy; SD, stable disease; SOC, standard-of-care; TMZ, temozolomide; VP, ventriculoperitoneal.

* All received GsONC201 as first-line therapy.

Fractionated RT was prescribed for 23 (85%) patients of which 17 (63%) had concomitant TMZ. Fifteen percent (4/27) of patients received symptomatic care due to their poor postoperative functional performance status or rapid disease progression. More than a third of patients received GsONC201 (37%, 10/27) and 8 (80%) started treatment as first-line monotherapy after RT (GsONC201 + SOC) (Table 1). The median duration of GsONC201 therapy was 7.4 months (range: 0.5-61.4). No GsONC201-associated adverse effects were observed.

For the entire cohort, 70% (19/27) patients developed tumor recurrence and 12 (44%) received second-line treatment. The commonest second-line treatment prescribed was bevacizumab (67%, 8/12) and the proportion of patients receiving this agent were comparable between the GsONC201 + SOC and SOC-alone groups (chi-square test, *P* value: .45). The median duration from tumor recurrence to any second-line systemic treatment was 5.6 weeks (range: 1 day to 23.6 weeks) and there was no distinct difference between the groups (*P* value: .67).

**Figure 1.**
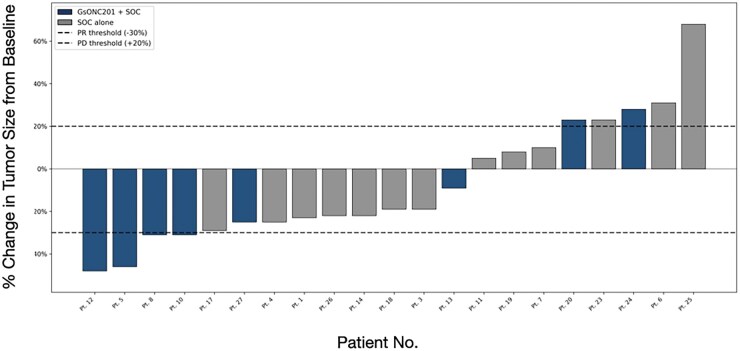
Waterfall plot of H3K27M-altered DMG size from baseline to 3 months after radiotherapy according to RECIST 1.1 criteria. A comparison between GsONC201 + SOC *versus* SOC-alone patients (*n* = 21). N.B. SOC, standard-of-care; PR, partial response; PD, progressive disease.

**Figure 2.**
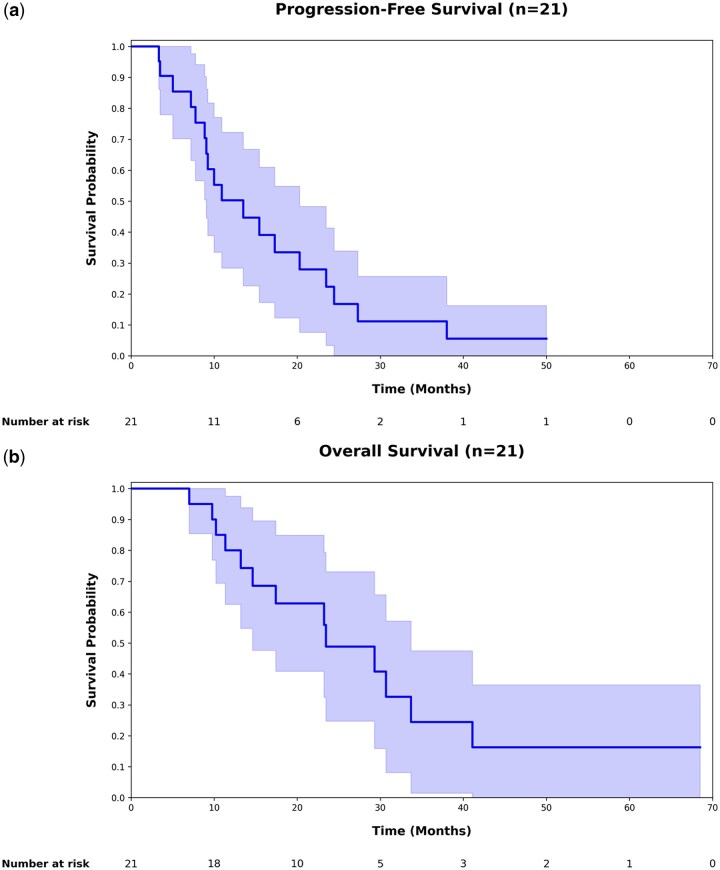
Kaplan-Meier survival curves for progression free survival (a) and overall survival (b) of H3K27M-altered DMG patients that completed standard-of-care fractionated radiotherapy.

**Figure 3.**
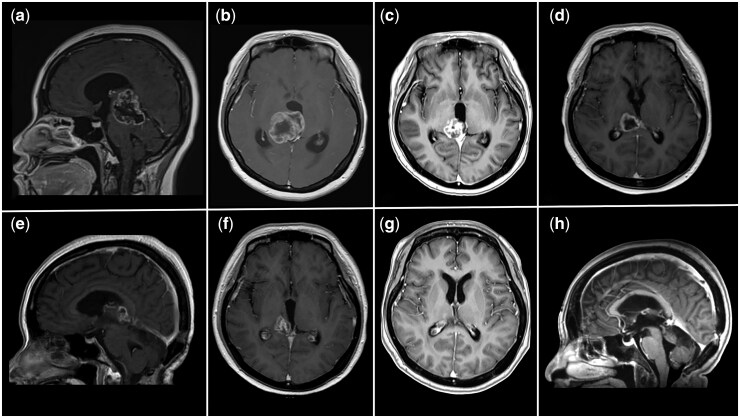
Illustrative case of a H3K27M-altered DMG patient experiencing sustained treatment response to first-line GsONC201 monotherapy after fractionated radiotherapy. A 31-year-old woman presented with progressive drowsiness and headache over 2 weeks with a presenting gadolinium contrast-enhanced MRI scan revealing a right thalamic intra-axial heterogeneously enhancing tumor causing obstructive hydrocephalus (a, sagittal; b, axial). Craniotomy for partial resection and fractionated radiotherapy were performed (60Gy/30Fr). A post-treatment MRI scan 3 months after the operation revealed interval tumor regression with resolution of hydrocephalus (c). The patient was then prescribed oral GsONC201, 625 mg weekly, 15 weeks after her initial operation and 4 weeks after radiotherapy. An interim scan performed after 4 months of GsONC201 exhibited cystic degeneration of the tumor (d). After 9 months of GsONC201 monotherapy, a partial response according to RANO 2.0 criteria was observed (e, sagittal; f, axial). A subsequent scan after 24 months of GsONC201 demonstrated complete response (g, axial; h, sagittal). The patient continues to take the targeted therapy without any significant adverse effects and with a functional performance status of ECOG 0. Her overall survival is > 65 months (5.2 years).

Twenty-one (78%) patients completed SOC and had follow-up assessments at three months post-RT (Table 2 and Figure 1). According to RANO 2.0 criteria, the ORR was 33% (7/21). Fifteen percent (2/13) of SOC-alone patients and 63% (5/8) of GsONC201 + SOC patients experienced a RANO 2.0-defined minor treatment response (HR: 9.2; 95% CI: 1.1-73.2). For the five GsONC201 patients that had a response, the median duration from GsONC201 treatment start date to radiological response was 27 weeks (IQR: 22-32). 14% (3/21) patients experienced tumor recurrence (Table 3). Fifteen percent (2/13) of SOC patients had disease progression compared to 13% (1/8) of GsONC201 + SOC patients (*P* value: .10) (Table 2). At three months post-RT, according to RECIST 1.1 assessment, 62% (13/21) patients had a mean tumor size reduction of 27 ± 11% (Figure 1).  The mean change in size for GsONC201 patients was −17 ± 29% and for SOC-alone patients, it was −1.1 ± 29% (Mann-Whitney *U* test, *P* value: .12). Among the treatment responders, the mean tumor size reduction was 31 ± 14% for GsONC201 + SOC patients compared to a mean reduction of 23 ± 3% for SOC-alone patients (Mann-Whitney *U* test, *P* value: .14). Significantly more GsONC201 patients experienced an MRI-observable treatment response (Table 2). mPFS for the patients that completed RT was 9.2 months (IQR: 5.0-18.8). For GsONC201 + SOC patients, the mPFS was 13.5 months (IQR: 2.6-38.0) compared to 8.1 months (IQR: 4.1-16.7) for SOC-alone patients (Table 2 and Figure 2). The mOS for the entire cohort was 14.6 months (IQR: 11.3-26.4). For those that completed RT, the mOS was 17.4 months (IQR: 12.1-30.0) (Table 2). The mOS of GsONC201 + SOC patients was 18.9 months (IQR: 11.3-54.4) compared to 16.0 months (IQR: 12.5-27.6) for SOC-alone patients (Figure 2). Due to the small sample size, the study was underpowered to analyze predictors for PFS and OS, particularly regarding the role of first-line GsONC201. A larger sample size would be needed to conduct multivariate modelling or propensity-score analysis. A H3K27M-altered DMG patient who received RT with first-line GsONC201 monotherapy and experienced complete tumor remission with more than five years of OS is described (Figure 3).

**Table 2.** Outcomes of H2K7M-mutant DMG patients: comparison of SOC-alone patients *versus* GsONC201 + SOC patients^*^

Outcomes	Entire cohort	SOC-alone	GsONC201 + SOC^*^	*P* value
	*n* = 21 (%)^*^	*n* = 13 (%)	*n* = 8 (%)	
Recurrence	21 (100)	13 (100)	8 (100)	-
RANO 2.0 response category at 3 months post-RT^*^				
CR	0	0	0	-
PR ≥ 50% ↓	0	0	0	-
MR 25-50% ↓	7 (33)	2 (15)	5 (63)	.03
SD < 25% ↓	11 (52)	9 (69)	2 (25)	.05
PD > 25% ↑ or new lesion	3 (14)	2 (15)	1 (13)	.10
RECIST 1.1 response category at 3 months post-RT				
CR	0	0	0	-
PR ≥ 30% ↓	4 (19)	0	4 (50)	.01
SD < 30% ↓	12 (57)	10 (77)	2 (25)	.02
PD ≥ 20% ↑ or new lesion	5 (24)	3 (23)	2 (25)	.92
PFS, months, median (IQR)	9.2 (5.0-18.8)	8.1 (4.1-16.7)	13.5 (8.9-38.0)	.10
PFS 6 months	17 (63)	9 (69)	8 (100)	.28
OS, months, median (IQR)	17.4 (12.1-30.0)	16.0 (12.5-27.6)	18.9 (11.3-54.4)	.57
OS 6 months	21 (100)	13 (100)	8 (100)	.31
12 months	16 (76)	11 (85)	5 (63)	.26
18 months	10 (48)	6 (46)	4 (50)	.49

CR, complete remission; GsONC201, German-sourced ONC201; MR, minor response; OS, overall survival; PD, progressive disease; PFS, progression-free survival; PR, partial remission; RANO, radiological assessment in neuro-oncology; RECIST, response evaluation criteria in solid tumors; RT, radiotherapy; SD, stable disease; SOC, standard-of-care.

* Twelve patients reviewed since 4 patients received symptomatic treatment after diagnosis and 2 defaulted follow-up.

**Table 3.** Clinical, molecular, treatment, and outcomes of adult Chinese H3K27M-mutant diffuse midline glioma patients

Patient no.	Age (years)/Sex	Preop ECOG	Location	Other molecular features^§^	EOR	First-line treatment	RANO 2.0 Category 3 months post-RT	RECIST 1.1 Category 3 months post-RT (change in tumor size form baseline)	Recurrence	PFS (months)	Second-line treatment	Duration of GsONC201 (months)	Current status	OS (months)
1	33/F LCY	2	Pons	ATRX preserved *p53* mutation -	Biopsy	TMZ CRT (60Gy/30Fr)	SD	PD (-23%)	Yes	10.9	None	-	NA	28.0
2	29/F KTY	2	Thalamus	ATRX preserved *p53* mutation + *BRAF^V600E^* mutation -	STR	Symptomatic	NA	NA	NA	NA	None	-	Dead	1.2
3	38/M LMY	1	Thalamus	ATRX loss *p53* mutation +	STR	TMZ CRT (60Gy/30Fr)	SD	SD (-19%)	Yes	7.8	TTF		Dead	9.8
4	21/F CHT	1	Pons	*PIK3CA* amp + *SOX2* amp + *PRKCI* amp + *BCL6* amp + *EGFR* amp -	Biopsy	TMZ CRT (50.4Gy/28 Fr)	MR	SD (-25%)	Yes	10.0	Bevacizumab + CCNU	-	Dead	14.6
5	19/F 21 LCC	0	Pons		Biopsy	TMZ CRT (54Gy/30 Fr)GsONC201	MR	PR (-46%)	Yes	9.2	Bevacizumab	3.0	Dead	11.3
6	37/M LLY	1	Thalamus	*H3F3A*	STR	TMZ CRT (60Gy/30Fr)	PR	PR (+31%)	Ye	20.3	None	-	Dead	23.5
7	73/F CSL	2	Hypothalamus	*H3F3A* mutation (c.83A>T, p. Lys28Met) ATRX preserved *EGFR* amp -	Biopsy	RT (34Gy/10Fr)	SD	SD (+10%)	Yes	3.5	None	-	Dead	23.2
8	31/F LHI	2	Thalamus	ATRX loss	STR	RT (54Gy/30Fr) → GsONC201	MR	PR (-31%)	Yes	38.0	SRS + GsONC201	61.4	Alive	64.8
9	25/F LTY	1	Thalamus	ATRX preserved *p53* mutation +	Biopsy	TMZ CRT (54Gy/30 Fr)	NA	NA	NA	NA	NA	-	NA	NA
10	51/F HLK	0	Thalamus	ATRX preserved *EGFR* amp -	STR	TMZ CRT (60Gy/30 Fr) → GsONC201	MR	PR (-31%)	Yes	8.9	None	3.10	Dead	10.2
11	52/M NW	3	Thalamus	*H3F3A* mutation (c.83A>T, p. Lys28Met) ATRX loss BRAF^*V600E*^ mutation -	Biopsy	TMZ CRT (60Gy/30 Fr)	SD	SD (+5%)	Yes	23.5	None	-	Dead	30.7
12	42/F CPY	3	Thalamus	*H3F3A* mutation (c.83A>T, p. Lys28Met) ATRX loss *p53* mutation - *EGFR* amp -	STR	RT (60Gy/30Fr) → GsONC201	MR	PR (-48%)	No	-	Bevacizumab + irinotecan	14.9	Alive	46.8
13	45/F LOY	0	Medulla		Biopsy	TMZ CRT (45Gy/25Fr) → GsONC201	SD	SD (-9%)	Yes	17.3	Bevacizumab	17.2	Dead	29.3
14	52/M CHL	2	Thalamus	*H3F3A* mutation (c.83A>T, p. Lys28Met) ATRX preserved *TERT* mutation - *EGFR* amp -	STR	RT (34Gy/10 Fr) → TMZ	SD	SD (-22%)	Yes	15.4	None	-	Dead	17.4
15	40/F THY	1	Hypothalamus	*H3F3A* mutation (c.83A>T, p. Lys28Met) ATRX preserved *p53* mutation + *EGFR* amp - PTEN loss - *BRAF^V600E^* mutation -	STR	TMZ CRT (60Gy/30Fr)	NA	NA	Yes	24.4	Bevacizumab + irinotecan	-	Dead	41.1
16	19/F TLJ	2	Pons	*H3F3A* mutation (c.83A>T, p. Lys28Met) ATRX preserved *p53* mutation + *BRAF^V600E^* mutation -	STR	Symptomatic	NA	NA	NA	-	None	-	Dead	2.6
17	30/F LWH	0	Hypothalamus	*H3F3A* mutation (c.83A>T, p. Lys28Met) *p53* mutation +	STR	TMZ CRT (54Gy/30Fr)	MR	SD (-29%)	Yes	5.0	GsONC201 + TTF	0.5	Dead	7.0
18	41/M LYF	3	Thalamus	*H3F3A* mutation (c.83A>T, p. Lys28Met) ATRX preserved *p53* mutation - EZHIP -	STR	TMZ CRT (60Gy/30Fr)	SD	SD (-19%)	Yes	9.1	None	-	Dead	13.2
19	32/M LMT	1	Thalamus	ATRX loss *p53* mutation -	Biopsy	RT (50.4Gy/28 Fr)	SD	SD (+8%)	Yes	27.3	TMZ	-	Dead	33.7
20	60/M CTS	3	Medulla		Biopsy	TMZ CRT (54Gy/30Fr) → GsONC201	SD	SD (+23%)	Yes	8.5	RT (20Gy/5Fr) to cervical cord metastasis Bevacizumab + CCNU	30.1	Dead	11.3
21	36/M WKM	2	Thalamus	ATRX preserved *p53* mutation +	STR	Symptomatic	NA	NA	NA	NA	None	-	Dead	2.0
22	36/F CTW	3	Pons	*H3F3A* mutation (c.83A>T, p. Lys28Met) ATRX preserved	Biopsy	Symptomatic	NA	NA	NA	NA	None	-	Dead	6.6
23	70/M CTL	2	Thalamus	*H3F3A* mutation (c.83A>T, p. Lys28Met) ATRX preserved *p53* mutation + *TERT* mutation—*EGFR* amp -	STR	TMZ CRT (40Gy/15 Fr)	SD	PD (+23%)	Yes	7.2	Bevacizumab + TTF	-	Dead	12.9
24	48/M CCM	1	Thalamus	*H3F3A* mutation (c.83A>T, p. Lys28Met)	Biopsy	TMZ CRT (60Gy/30 Fr) → GsONC201	PD	PD (+28%)	Yes	3.4	GsONC201	11.4	Alive	17.8
25	56/F WMS	0	Pons	ATRX preserved *p53* mutation + *TERT* mutation—*EGFR* amp - PTEN loss -	Biopsy	TMZ CRT (54Gy/30 Fr)	PD	PD (+68%)	No	13.5 MR	None	13.3 13.5	Alive	18.9
26	56/M CMY	2	Midbrain		Biopsy	RT (54Gy/30 Fr)	SD	SD (-22%)	No	10.9	None	-	Dead	13.8
27	47/F CWM	1	Thalamus	*H3F3A* mutation (c.83A>T, p. Lys28Met)	STR	TMZ CRT (60Gy/30 Fr) → GsONC201	MR	SD (-25%)	No	9.0	None	3.5	Alive	13.0

CRT, chemoradiotherapy; ECOG, Eastern Cooperative Oncology Group; GsONC201, German-sourced ONC201; RT; radiotherapy; SRS, stereotactic radiosurgery; STR, subtotal resection; TMZ, temozolomide; TTF, tumor-treating fields therapy.

§ All tumor specimens were IHC immune-positive for H3K27M ± immuno-negative for H3K27me3. Additional histone H3 Sanger sequencing results for H3-3A and/or H3SC gene mutations are reported if performed. All tumors were *IDH-1* wildtype and p*MGMT* unmethylated. None of the tumors were subject to next-generation sequencing due to public healthcare system resource constraints.

## Discussion

The exact incidence of H3K27M-altered DMG among Chinese adults is unknown. Hong Kong delivers universal healthcare that demands the reporting of all histologically confirmed high-grade glioma diagnoses. Since most patients would eventually require public hospital institutional support, it was believed that patient identification was near-complete and a minimal number of patients were missed. The crude incidence of H3K27M-altered DMG among Chinese adults was 0.04 per 100 000 and is comparable with the results from the US National Cancer Institute’s Surveillance, Epidemiology and End Results database that cites an incidence of 0.06 per 100 000.^2^

This study is the only existing report on the outcomes of Chinese patients using imipridone class agents for this rare tumor with the longest duration of GsONC201 administration and clinical follow-up in the biomedical literature. We demonstrated that DRD2 antagonist treatment was well-tolerated and corroborates the findings of real-world studies that reviewed largely pediatric age-group patients.^7^^,^^8^^,^^14^^,^^15^ However, given the limited sample size, our study was under-powered to draw meaningful conclusions on the effect of GsONC201 on OS.

H3K27M-altered DMG is notoriously difficult to treat and although significant progress was made in their molecular classification, therapeutic development has remained stagnant despite numerous clinical trials.^5^ Before the introduction of the WHO’s integrated multilayered approach, a presumptive diagnosis of diffuse intrinsic pontine glioma (DIPG) was frequently derived from imaging alone and neurosurgeons were less inclined to biopsy, much less resect, these lesions. The only existing clinical practice guideline for DMG management recommends that maximal safe resection should be attempted when feasible or at least a biopsy of midline intrinsic tumors to confirm the presence of H3K27M alterations.^21^ The cornerstone of management is fractionated RT, but it confers a mOS of only 9-11 months and a two-year survival rate of less than 10%.^5^^,^^21–23^

No effective systemic agent has been proven effective. This is largely due to the blood brain barrier where intratumoral drug levels are often insufficient to elicit significant cytotoxicity. In addition, our study supports the findings of others that H3K27M-altered DMGs possess intrinsic chemoresistance owing to their high MGMT expression and proficient DNA repair pathways.^24^ These observations have driven the exploration of novel agents and therapeutic strategies. The clinical trial landscape for H3K27M-altered DMGs is extensive and rapidly evolving, with over 40 active or recently completed interventional studies.^5^ These trials encompass epigenetic modulators, mutation-specific vaccines, chimeric antigen receptor (CAR) T-cell therapies, and poly(ADP-ribose) polymerase (PARP) inhibitors.^25–30^

Among these emerging therapies, ONC201 has garnered significant attention as a novel oral targeted therapy small molecule inhibitor. Its favorable safety profile and oral bioavailability make it a promising agent. Pharmacokinetic studies of glioblastoma tissue acquired during repeat resection for patients previously on ONC201 detected therapeutic intratumoral drug concentrations as soon as 24 h after administration.^31^ ONC201 selectively antagonizes DRD2 and activates mitochondrial ClpP, triggering an integrated stress response and inducing tumor cell apoptosis.^9^ The duration of GsONC201 administration is an important factor to consider. Mechanistically, ONC201 genomically restores onco-suppressive H3K27me3 by gradually disrupting metabolic and epigenetic pathways.^9–11^ ONC201-mediated mitochondrial depletion of TCA cycle oxoglutarate dehydrogenase results in the accumulation of α-ketoglutarate and 2-hydroxyglutarate levels to genotoxic levels.^9^ ONC201 also reduces chromatin accessibility causing the epigenetic downregulation of genes associated with neuroglial differentiation and cell-cycle progression.^9^ Another probable mechanism of action is its immunostimulatory role that was first identified in prostate and endometrial cancer patients.^32^ A late immune effector response was noted among these patients with prolonged ONC201 exposure with evidence of intratumoral NK cell infiltration associated with elevated levels of immune cytokines such as IL-6, IL-10, and TNF-α.^32^ The observed delayed response duration of six months noted among our GsONC201 patients could reflect this unique immune-mediated mechanism of action. Our findings are also supported by a cohort study of recurrent DMG patients that documented the median observed response time was 8.3 months (1.9-15.9).^33^

Several case series and open-label phase II trials suggest that ONC201 improves outcomes in newly diagnosed H3K27M-altered DMG patients with mOS reaching up to 21.7 months.^5–9^^,^^34^^,^^35^ A real-world study of 174 DMG patients that obtained ONC201 through an expanded access program observed an mOS of 19.6 months for adults.^8^ There are currently three RCTs investigating ONC201 for adults with H3K27M-altered DMG. The Pediatric Neuro-Oncology Consortium study, PNOC022 (NCT05009992) is a multi-national phase II trial recruiting pediatric and young adult patients of 2-39 years, subjecting them to various combinations of ONC201, the HDAC inhibitor panobinostat, and the PI3K/Akt inhibitor paxalisib.^35^ The BIOMEDE 2.0 (NCT05476939) is a multi-center, randomized, open-label, controlled phase III trial evaluating the efficacy of ONC201 for patients ≥ 6 months old with no upper age limit. Finally, the multi-center placebo-controlled phase III ACTION trial (NCT05580562) is evaluating whether ONC201 following RT will extend OS, but it excludes patients with DIPG or intramedullary spinal tumors.^13^

Results from high resolution mass spectroscopy, nuclear magnetic resonance spectroscopy and orthotopic PDX murine models, demonstrated GsONC201 shares the same chemical composition (C_24_H_26_N_4_O_1_) and molecular structure as the original ONC201 formulation.^14^ The investigators also revealed that GsON201 also had similar pharmacokinetic and pharmacodynamic properties.^14^ GsONC201 is the active angular isomer [3,4-e] of ONC201 with the main difference being that its formulation is a free base instead of an acidic salt.^14^ The advantage of the original ONC201 dihydrochloric salt formulation is its improved water solubility, but it was observed that gastric pH levels are sufficiently low that such differences would likely be immaterial.^14^ Two real-world case series studies concluded GsONC201 was well-tolerated and clinically effective resulting in a patient mOS of 18-20 months.^14^^,^^15^ However, both recruited pediatric patients with one focusing solely on DIPG.^14^^,^^15^ Although our study sample size was too limited to derive definitive conclusions on survival, we noted that a greater proportion of GsONC201 adult patients experienced a partial response at 3 months after RT compared to their control group counterparts.

There are several study limitations. First, the study sample size was not sufficiently powered to evaluate the clinical efficacy of GsONC201 on survival. However, given the rarity of H3K27M-altered DMG, the urgent need for therapeutic breakthroughs and the increasingly encouraging results from real-world studies, we believed it was prudent to report our findings for adult Chinese patients. Second, many neurosurgical centers do not regularly perform immediate post-operative MRI scans. Therefore, it was not possible to evaluate volumetric EOR and we could only resort to the neurosurgeon’s intraoperative assessment. This is especially relevant for thalamic DMGs that are more amenable to resection, but several have also concluded that EOR was unrelated to OS.^36–39^ Third, patients took GsONC201 at variable times since diagnosis. Although 80% received the agent as first-line therapy, the durations were spread over five months. This was largely a result of the logistical issues involved in importing GsONC201to Hong Kong during the COVID-19 pandemic. In comparison, for the ONC201-014 trial (NCT03416530), patients, received the agent as a median time of 1.8 months after RT.^9^ Few patients had their tumors subject to comprehensive molecular profiling and reflects the heterogeneity of real-world neuropathology practice across different laboratories in our locality. There is increasing evidence that H3K27M-altered DMGs are not homogeneous tumors. Patients with DMGs exhibiting loss of *ATRX* expression or *H3F3A* mutations, as opposed to *HIST1H3B* mutations, had a poorer prognosis.^40^^,^^41^ Finally, health-related quality-of-life (QoL) assessments were not performed which would have provided a more comprehensive assessment of the clinical utility of GsONC201. But given the observed safety of this agent and its once-weekly administration, it was believed that the impact on QoL was minimal.

H3K27M-altered DMG is a rare tumor and patients have a poor prognosis. We observed that first-line imipridone class DRD2 targeted therapy after RT was well-tolerated. However, its effect on OS remains to be determined by well-designed RCTs.

## Data Availability

The datasets analyzed during the current study are available from the corresponding author on reasonable request.
